# Evaluating the predictive value of log odds of positive lymph nodes on postoperative survival in patients with laryngeal cancer: a SEER population-based study

**DOI:** 10.1007/s12672-025-02193-z

**Published:** 2025-04-02

**Authors:** Jiahui Zhang, Wenjun Su, Yue Wang, Peiji Zeng, Wei Wang, Wenjie Fu, Chengfu Cai

**Affiliations:** 1 Xiamen Hospital of Traditional Chinese Medicine, Xiamen, China; 2 Department of Otolaryngology-Head and Neck Surgery, Zhongshan Hospital of Xiamen University, School of Medicine, Xiamen University, Xiamen, China; 3 School of Medicine, Xiamen University, Xiamen, China; 4 The School of Clinical Medicine, Fujian Medical University, Fuzhou, China; 5 ENT Institute and Otorhinolaryngology Department of Eye & ENT Hospital, State Key Laboratory of Medical Neurobiology and MOE Frontiers Center for Brain Science, Fudan University, Shanghai, China

**Keywords:** Laryngeal cancer, Prognostic model, LODDS, TNM staging

## Abstract

Traditionally, the AJCC TNM staging system has been the primary tool for assessing the severity and prognosis of laryngeal cancer. Although several studies have demonstrated that the log odds of positive lymph nodes (LODDS) offers superior predictive accuracy compared to the TNM staging for other cancers, there is limited research for laryngeal cancer. This study analyzed data from SEER database (2000–2019). Independent risk factors for survival were identified using univariate and multivariate Cox regression analyses, and different prognostic models were constructed based on the multivariate analysis results. The predictive performance of these models was evaluated using receiver operating characteristic (ROC) curves and area under the curve (AUC) values. The results indicated that LODDS subgroup, age, marital status, histologic grade, T-stage, and N-stage were consistent independent prognostic factors for overall survival (OS) and cancer-specific survival (CSS). Assessment metrics showed that the multivariate model, which incorporated both LODDS and N staging, outperformed the individual N staging and LODDS models in predicting postoperative prognosis in laryngeal cancer patients. Overall, the multivariate model constructed in this study is a superior tool for predicting the postoperative status of laryngeal cancer.

## Introduction

Laryngeal cancer is one of the most common malignancies in otolaryngology, surpassed in incidence only by papillary thyroid carcinoma. Each year, approximately 188,960 new cases of laryngeal cancer are diagnosed, representing 0.9% of all cancer cases, with 103,216 deaths attributed to the disease, which accounts for 1.1% of all cancer-related mortality [1]. Over 60% of laryngeal cancer patients are diagnosed at an advanced stage [2]. Laryngeal cancer comprises several subtypes based on the diverse origins of blastocyst clusters. Among these, the supraglottic and subglottic subtypes frequently involve cervical lymph node metastasis, with metastasis rates of 19.9% and 8.0%, respectively. [3, 4]. In contrast, the glottic subtype is less prone to lymph node metastasis due to the anatomical barrier of the elastic cone and limited lymphatic drainage in the glottic region. These distinct patterns of metastasis complicate the prediction of laryngeal cancer prognosis. Given the larynx’s critical functions in breathing, phonation, swallowing, and breath control [5], surgical treatment can lead to complications such as voice loss and dysphagia, significantly affecting postoperative quality of life [6, 7]. In addition to surgery, treatment strategies for laryngeal cancer often include radiotherapy, chemotherapy, and immunotherapy, either alone or in combination, making accurate prognosis assessment after surgery more challenging [8, 9].

The American Joint Committee on Cancer (AJCC) Tumor, Node, Metastasis (TNM) staging system remains the gold standard for evaluating the status and prognosis of cancer patients. In laryngeal cancer, N staging assesses regional lymph node involvement; however, it does not consider the exact count of positive lymph nodes [10]. The Log Odds of Positive Lymph Nodes (LODDS) is a metric derived from the number of positive lymph nodes (NPLN) and the number of dissected lymph nodes (NDLN), calculated using the formula ln([NPLN + 0.5]/[NDLN—NPLN + 0.5]). A higher LODDS value reflects an increased NPLN and is associated with a poorer prognosis [11]. LODDS has found extensive application in tumors affecting the digestive, respiratory, and urinary systems, with prognostic models based on LODDS often demonstrating superior predictive accuracy compared to the TNM staging system [12–14]. However, its application in laryngeal cancer remains relatively limited.

To develop more effective postoperative prediction models for laryngeal cancer patients, this study evaluates the predictive performance of LODDS-based models and compares their accuracy with the AJCC TNM N staging system, utilizing data from the Surveillance, Epidemiology, and End Results (SEER) database, which offers comprehensive demographic and clinical information on cancer patients in the United States.

## Materials and methods

### Data selection

**Fig. 1:**
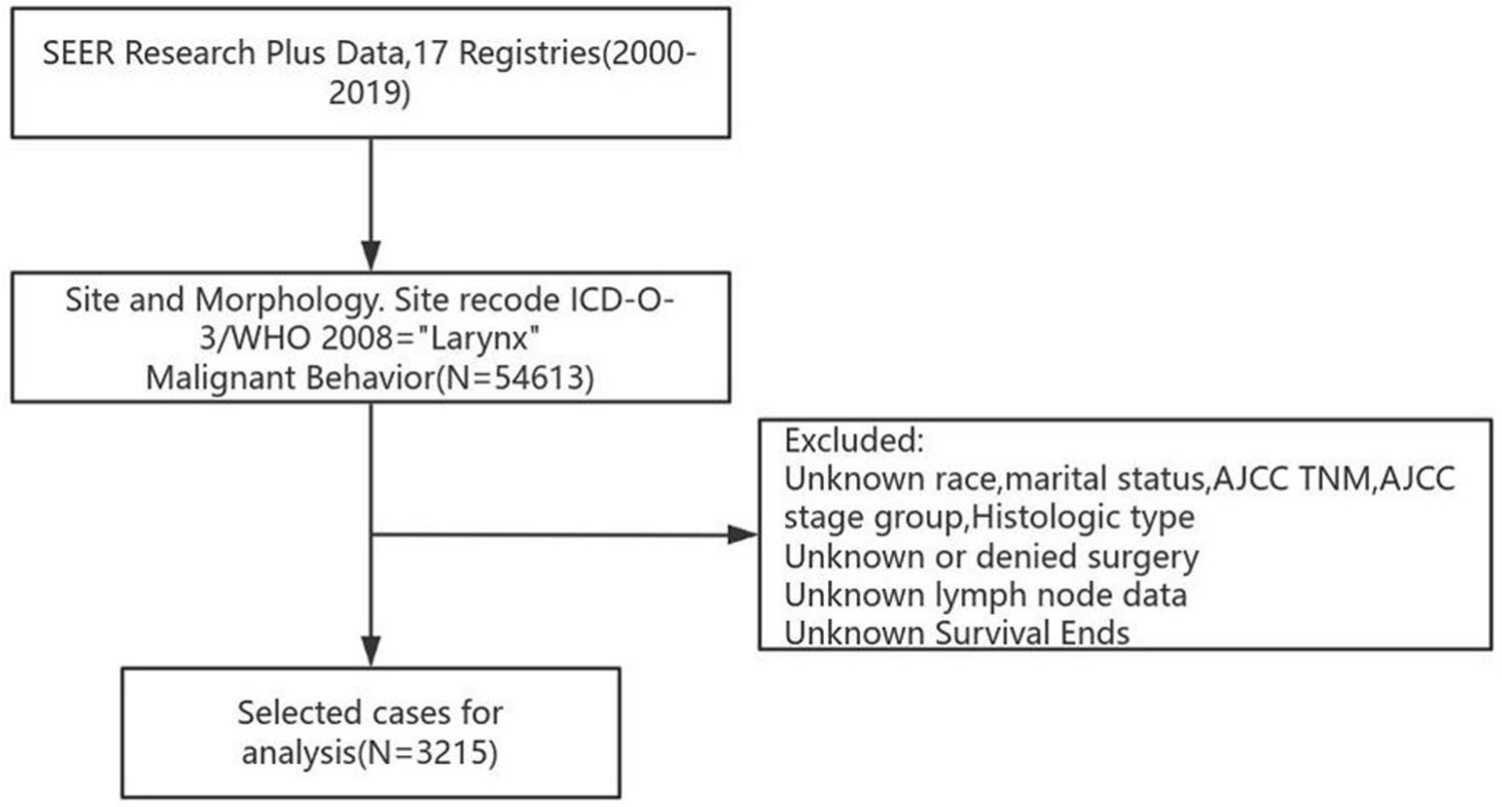
Flowchart of the data cleaning and screening process

The cases selected for this study were extracted from the SEER database (version 8.4.3), utilizing the “Incidence SEER 17 Registries Nov 2021 Sub” dataset covering the years 2000 to 2019. We specifically targeted cases categorized as “larynx” based on the International Classification of Diseases for Oncology, Third Edition (ICD-O-3), and the World Health Organization (WHO) 2008 coding for site and morphology, identifying a total of 54,613 malignant cases. The criteria for exclusion included the following: (1) cases with unknown race, marital status, derived AJCC T, N, M staging, derived AJCC stage group, or histologic type (ICD-O-3); (2) cases with unknown or denied surgical treatment; (3) cases lacking lymph node data; (4) cases with insufficient data on the NPLN; and (5) cases with unknown survival outcomes. The workflow is illustrated in Fig. 1.

### Variable selection and data handling

The dataset included the following extracted variables: “Age recode (including individuals under 1 year),” “Race recode (White, Black, Other),” “Sex,” “Marital Status,” “Primary Site (labeled),” “Grade (thru 2017),” “Derived AJCC T, 6th Edition (2004–2015),” “Derived AJCC N, 6th Edition (2004–2015),” “Derived AJCC Stage Group,” “Regional Nodes Positive (1988 +),” “Radiation Recode,” “Chemotherapy Recode (Yes, No/Unknown),” “Survival Months,” “SEER Cause-Specific Death Classification,” and “Vital Status Recode (study cutoff used).”

The variables selected for this study included age, sex, race, marital status, primary tumor site, histological grade, T stage, N stage, tumor stage, radiation therapy, and chemotherapy. Among these, “Age recode (including individuals under 1 year)” and the LODDS, calculated from the NPLN, were treated as continuous variables. Age was divided into two categories: “less than 60 years” and “60 years or older.”

The X-tile software, version 3.6.1, was employed to ascertain the optimal LODDS threshold values that maximized survival differences. This tool is widely recognized for its utility in determining optimal cut-off points in survival analyses and for processing continuous data derived from medical research [15]. The patients were stratified into three distinct groups based on the cutoff values.

This study defined overall survival (OS) as the primary endpoint and cancer-specific survival (CSS) as the secondary endpoint. OS, the primary outcome measure, represents the time from diagnosis to death, accounting for mortality resulting from both tumor-related and non-tumor-related causes. Conversely, CSS refers to the survival duration from diagnosis until death specifically attributable to cancer, excluding mortality from non-cancer-related causes.

### Statistical analysis

In this study, statistical analyses were conducted using R software (version 4.3.3) and SPSS (version 29). A p-value below 0.05 was regarded as statistically significant. Categorical variables were reported as frequencies (n) and percentages (%). Evaluations utilized the chi-square test and Fisher’s exact test. To identify independent risk factors for OS and CSS, both univariate and multivariate Cox regression analyses were conducted. Variables with p-values less than 0.05 were incorporated into the prognostic prediction model. Survival curves were plotted using Kaplan–Meier survival analysis, which was based on various classifications of LODDS and N staging. Receiver operating characteristic (ROC) curves evaluated the prognostic predictive value of the multivariate, LODDS, and N staging models by comparing the area under the curve (AUC) for each. Ultimately, a nomogram was employed to visualize the optimal predictive model, enhancing the interpretation of prognostic predictions.

## Results

### Demographic and clinicopathological characteristics

After the screening process, this study included a total of 3,215 cases. Using the X-tile software, we identified LODDS intercept values of − 1.46 and − 0.91. Based on these threshold values, patients were classified into three groups: Group 1 (LODDS < − 1.46), Group 2 (− 1.46 ≤ LODDS < − 0.91), and Group 3 (LODDS ≥ − 0.91). As presented in Table 1, The three groups exhibited significant differences regarding the primary tumor site, histological grade, N stage, tumor stage, and the use of radiotherapy and chemotherapy. Group 3 patients were more likely to have tumors situated in the supraglottic region, while Group 1 had a higher proportion of glottic tumors. In terms of histological grade, patients in Group 3 generally exhibited poorer differentiation compared to those in Groups 1 and 2. Regarding lymph node metastasis, patients in Groups 2 and 3 had a higher likelihood of metastasis and advanced tumor stage compared to Group 1. The use of adjuvant therapies such as radiotherapy and chemotherapy was more prevalent in Groups 2 and 3 than in Group 1. Additionally, This analysis indicated that age, gender, race, marital status, and T stage exhibited no significant differences among the three groups.

**Table 1:** Baseline characteristics of patients with laryngeal cancer undergoing surgery

Variables	Lodds < − 1.46 (n = 1404)	Lodds-1.46 ≤ Lodds < − 0.91 (n = 864)	Lodds ≥ − 0.91 (n = 947)	P
Age				0.399
< 60 years old	629 (44.8%)	373(43.2%)	398(42.0%)	
≥ 60 years old	775 (55.1%)	491(56.8%)	549(58.0%)	
Sex				0.374
Male	1137 (80.9%)	715(82.8%)	760(80.3%)	
Female	267 (19.0%)	149(17.2%)	187(19.7%)	
Race				0.343
White	1120 (79.7%)	675(78.1%)	743(78.5%)	
Black	232 (16.5%)	141(16.3%)	160(16.9%)	
Other	52 (3.7%)	48(5.6%)	44(4.6%)	
Marital status				0.837
Married	695(49.5%)	419(48.5%)	449(47.4%)	
Unmarried	359(25.5%)	217(25,1%)	252(26.6%)	
Other	350(24.9%)	228(26.4%)	246(26.0%)	
Primary site				< 0.001
Supraglottis	577(41.1%)	407(47.1%)	456(48.2%)	
Glottis	528(37.6%)	253(29.3%)	264(27.9%)	
Subglottis	50(3.5%)	34(3.9%)	39(4.1%)	
Other	249(17.7%)	170(19.7%)	188(19.9%)	
Differentiation				< 0.001
Grade I	140(10%)	49(5.7%)	67(7.1%)	
Grade II	899(64.0%)	484(56.0%)	494(52.2%)	
Grade III	353(25.1%)	325(37.6%)	377(39.8%)	
Grade IV	12(0.9%)	6(0.7%)	9(1.0%)	
T				0.531
T1	139(9.9%)	72(8.3%)	92(9.7%)	
T2	215(15.3%)	120(13.9%)	143(15.1%)	
T3	363(25.9%)	210(24.3%)	233(24.6%)	
T4	687(48.9%)	462(53.5%)	479(50.6%)	
N				< 0.001
N0	1152(82.0%)	193(22.3%)	225(23.8%)	
N1	183(13.0%)	220(25.5%)	164(17.3%)	
N2	67(4.7%)	431(49.9%)	531(56.1%)	
N3	3(0.2%)	20(2.3%)	27(2.9%)	
Stage				< 0.001
I	122(8.7%)	27(3.1%)	34(3.6%)	
II	179(12.7%)	27(3.1%)	37(3.9%)	
III	370(26.4%)	154(17.8%)	141(14.9%)	
IV	733(52.2%)	656(75.9%)	735(77.6%)	
Radiotherapy				< 0.001
Yes	785(55.9%)	582(67.4%)	658(69.5%)	
None/unknown	619(44.1%)	282(32.6%)	289(30.5%)	
Chemotherapy				< 0.001
Yes	257(18.3%)	319(36.9%)	402(42.4%)	
None/unknown	1147(81,7%)	545(63.1%)	545(57,6%)	

### Survival analysis

**Fig. 2:**
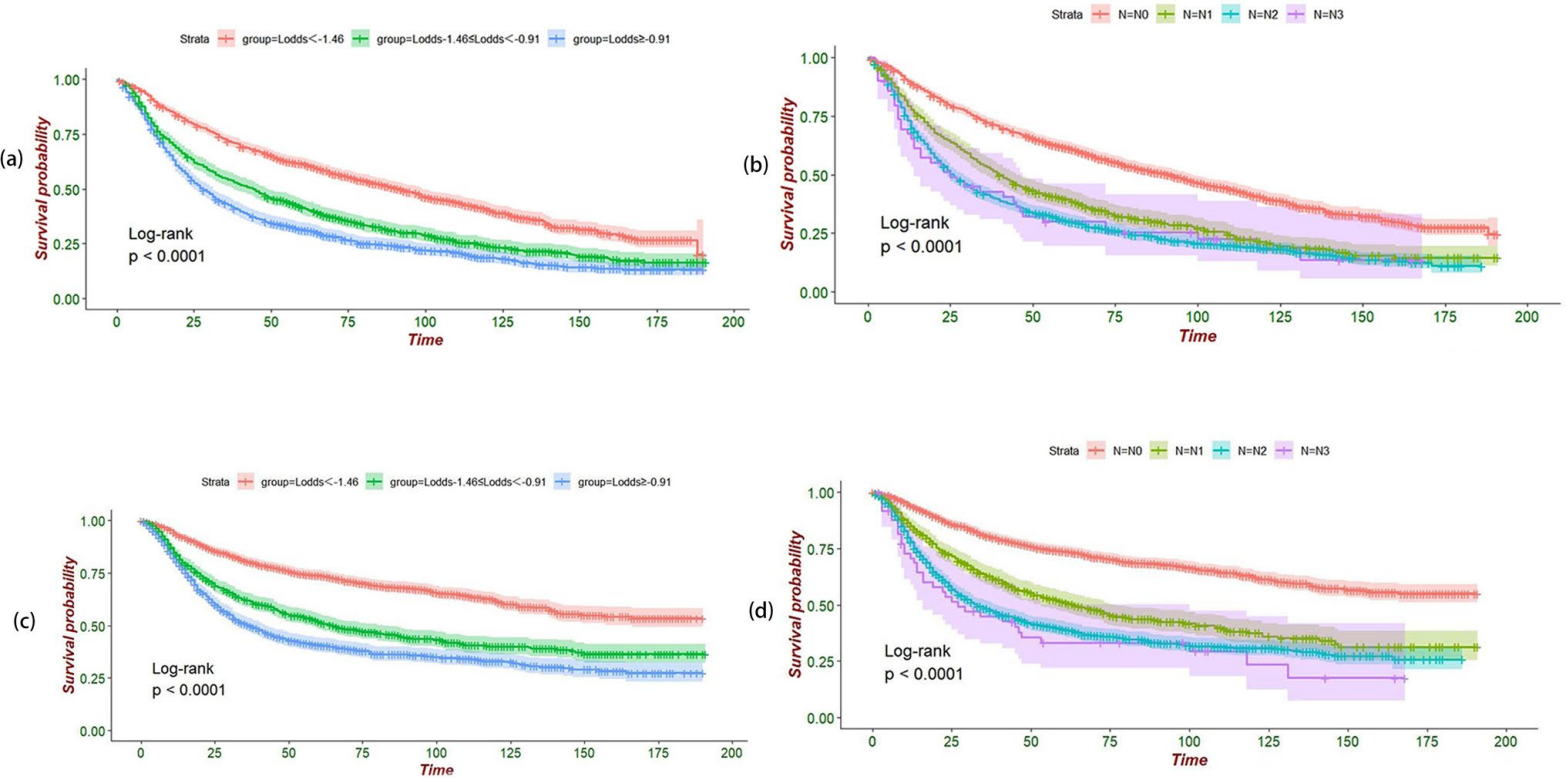
Kaplan–Meier curves of survival rates for patients in LODDS and N stage groups. **a** LODDS groups for OS; **b** N stage groups for OS; **c** LODDS groups for CSS; **d** N stage groups for CSS

Kaplan–Meier survival analysis was conducted for OS and CSS based on LODDS and N staging. The overall median survival time was 53 months (48.98–57.92). For LODDS staging (Fig. 2a), The median survival times for the individual groups were as follows: 94 months (83.73–100.28) for Group 1, 44 months (37.78–50.22) for Group 2, and 28 months (25.09–30.91) for Group 3. These results suggest that Group 1 had the most favorable survival outcomes, with Group 2 showing better survival than Group 3. Additionally, the OS survival curves differed significantly between the groups (p < 0.001), with distinct separations observed between each group’s curves. Regarding N staging (Fig. 2b), the median survival times were 92 months (84.37–99.63) for group N0, 40 months (34.91–45.09) for group N1, 26 months (23.36–28.64) for group N2, and 25 months (11.31–38.69) for group N3. The survival curves for this staging method differed statistically (p < 0.001); however, substantial overlap was observed among the curves for the N1, N2, and N3 groups. Similar results can be observed in CSS (Fig. 2c and Fig. 2d).

### Univariate and multivariate cox regression analysis

Univariate and multivariate Cox regression analyses identified independent risk factors for OS and CSS (Tables 2 and 3). For OS, the univariate regression analysis indicated that the following factors were significant risk factors: LODDS subgroups, age, gender, marital status, primary tumor site, histological grading, T stage, N stage, tumor stage, and chemotherapy. However, the multivariate regression analysis revealed that the primary tumor site was not an independent risk factor (Table 2). In terms of CSS, the univariate regression analysis identified LODDS subgroups, age, race, marital status, primary tumor site, histological grading, T stage, N stage, tumor stage, radiation therapy, and chemotherapy as potential risk factors. Nevertheless, the multivariate regression analysis did not demonstrate statistically significant differences for race, primary tumor site, tumor stage, and chemotherapy(Table 3).

**Table 2:** Analyses of overall survival in patients with laryngeal cancer undergoing surgery using both univariate and multivariate regression

Variables	Univariable analysis		Multivariable analysis	
	HR (95%CI)	P	HR (95%CI)	P
LODDS				
Lodds < − 1.46	Reference		Reference	
Lodds− 1.46 ≤ Lodds < -0.91	1.65(1.49–1.84)	< 0.001	1.19(1.05–1.34)	0.006
Lodds ≥ − 0.91	2.10(1.90–2.32)	< 0.001	1.56(1.38–1.76)	< 0.001
Age				
< 60 years old	Reference		Reference	
≥ 60 years old	1.58(1.44–1.72)	< 0.001	1.54(1.40–1.68)	< 0.001
Sex				
Male	Reference		Reference	
Female	0.89(0.80–0.99)	0.037	0.83(0.74–0.94)	0.002
Race				
White	Reference		Reference	
Black	1.07(0.96–1.20)	0.232	N/A	
Other	1.06(0.86–1.30)	0.596	N/A	
Marital status				
Married	Reference		Reference	
Unmarried	1.04(0.94–1.16)	0.412	1.10(0.99–1.22)	0.089
Separated/divorced/widowed	1.35(1.22–1.49)	< 0.001	1.30(1.17–1.44)	< 0.001
Primary site				
Supraglottis	Reference		Reference	
Glottis	0.88(0.79–0.97)	0.008	0.97(0.87–1.07)	0.519
Subglottis	1.11(0.89–1.38)	0.347	1.10(0.87–1.38)	0.430
Other	1.11(0.99–1.24)	0.078	1.06(0.93–1.19)	0.387
Differentiation				
Grade I	Reference		Reference	
Grade II	1.40(1.18–1.68)	< 0.001	1.24(1.03–1.48)	0.02
Grade III	1.76(1.47–2.11)	< 0.001	1.35(1.12–1.63)	0.002
Grade IV	1.79(1.12–2.86)	0.015	1.51(0.94–2.44)	0.089
T				
T1	Reference		Reference	
T2	1.26(1.05–1.52)	0.015	1.36(1.04–1.77)	0.023
T3	1.380(1.16–1.64)	< 0.001	1.36(1.07–1.74)	0.013
T4	1.63(1.39–1.91)	< 0.001	1.69(1.32–2.17)	< 0.001
N				
N0	Reference		Reference	
N1	1.76(1.57–1.98)	< 0.001	1.51(1.32–1.74)	< 0.001
N2	2.19(1.99–2.41)	< 0.001	1.82(1.56–2.12)	< 0.001
N3	2.20(1.59–3.03)	< 0.001	2.06(1.45–2.92)	< 0.001
Stage				
I	Reference		Reference	
II	1.06(0.81–1.38)	0.675	0.75(0.52–1.10)	0.144
III	1.52(1.21–1.90)	< 0.001	0.93(0.67–1.28)	0.159
IV	2.09(1.70–2.58)	< 0.001	0.82(0.59–1.14)	0.243
Radiotherapy				
Yes	Reference		Reference	
None/unknown	1.02 (0.93–1.11)	0.678	N/A	
Chemotherapy				
Yes	Reference		Reference	
None/unknown	0.81(0.74–0.88)	< 0.001	1.14(1.03–1.25)	0.009

**Table 3:** Analyses of cancer-specific survival in patients with laryngeal cancer undergoing surgery using both univariate and multivariate regression

Variables	Univariable analysis		Multivariable analysis	
	HR (95%CI)	P	HR (95%CI)	P
LODDS				
Lodds < − 1.46	Reference		Reference	
Lodds− 1.46 ≤ Lodds < -0.91	2.00(1.75–2.28)	< 0.001	1.26(1.08–1.47)	0.004
Lodds ≥ − 0.91	2.65(2.33–3.00)	< 0.001	1.72(1.48–2.01)	< 0.001
Age				
< 60 years old	Reference		Reference	
≥ 60 years old	1.39(1.25–1.55)	< 0.001	1.35(1.21–1.50)	< 0.001
Sex				
Male	Reference		Reference	
Female	0.88(0.77–1.01)	0.067	N/A	
Race				
White	Reference		Reference	
Black	1.09(0.93–1.25)	0.203	1.07(0.93–1.23)	0.373
Other	1.27(1.00–1.60)	0.047	1.14(0.90–1.45)	0.273
Marital status				
Married	Reference		Reference	
Unmarried	1.05(0.93–1.20)	0.424	1.05(0.92–1.21)	0.438
Separated/divorced/widowed	1.33(1.17–1.50)	< 0.001	1.24(1.09–1.40)	0.001
Primary site				
Supraglottis	Reference		Reference	
Glottis	0.86(0.76–0.97)	0.018	1.05(0.92–1.20)	0.452
Subglottis	1.00(0.75–1.32)	0.984	1.06(0.80–1.42)	0.675
Other	1.13(0.98–1.30)	0.082	1.10(0.95–1.28)	0.189
Differentiation				
Grade I	Reference		Reference	
Grade II	1.66(1.31–2.11)	< 0.001	1.41(1.10–1.79)	0.006
Grade III	2.19(1.71–2.80)	< 0.001	1.55(1.21–1.99)	0.001
Grade IV	2.72(1.60–4.61)	< 0.001	2.20(1.29–3.78)	0.004
T				
T1	Reference		Reference	
T2	1.43(1.12–1.83)	0.004	1.45(1.05–2.01)	0.007
T3	1.53(1.22–1.91)	< 0.001	1.44(1.07–1.95)	0.016
T4	1.95(1.58–2.41)	< 0.001	1.97(1.45–2.67)	< 0.001
N				
N0	Reference		Reference	
N1	2.10(1.82–2.43)	< 0.001	1.81(1.53–2.16)	< 0.001
N2	2.90(2.57–3.26)	< 0.001	2.32(1.92–2.80)	< 0.001
N3	3.40(2.40–4.81)	< 0.001	3.05(2.07–4.49)	< 0.001
Stage				
I	Reference		Reference	
II	1.20(0.83–1.75)	0.333	0.87(0.53–1.43)	0.592
III	1.83(1.33–2.52)	< 0.001	1.05(0.68–1.62)	0.817
IV	2.91(2.16–3.92)	< 0.001	0.94(0.61–1.47)	0.795
Radiotherapy				
Yes	Reference		Reference	
None/unknown	0.89(0.80–0.99)	0.036	1.34(1.18–1.52)	< 0.001
Chemotherapy				
Yes	Reference		Reference	
None/unknown	0.72(0.64–0.80)	< 0.001	1.01(0.89–1.15)	0.854

### Constructing and evaluating survival models

**Fig. 3:**
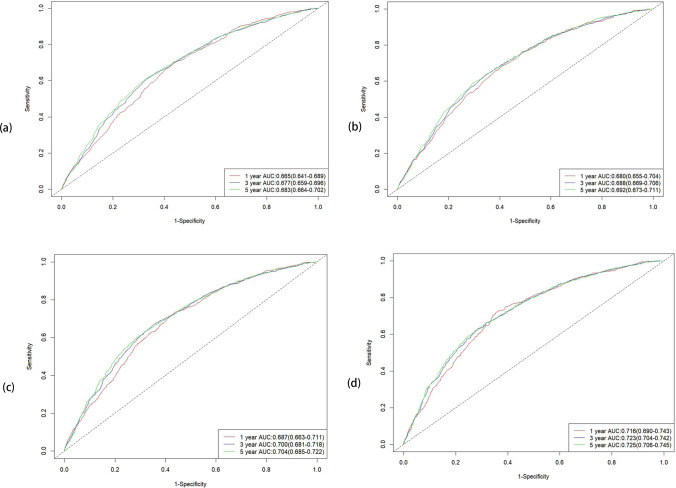
Time dependent ROC Curve shows the time-dependent ROC curve of model for predicting1-, 3- and 5-year survival rates of laryngeal cancer patients, The ROC curve provides the performance of the model under different discriminant thresholds. **a** LODDS model for OS, **b** N stage mode for OS, **c** multivariable model for OS, **d** multivarinble model for CSS

Based on the results of the multivariate Cox regression analysis, three predictive models were constructed: the LODDS model, the N staging model, and a combined model integrating both LODDS and N staging (referred to as the multivariate model). All three models, in addition to incorporating different staging methods, included four variables screened in a multivariate COX regression subcategorization: age, marital status, T stage, and histological grading. The accuracy and predictive abilities of these models were compared using ROC curves and AUC metrics. The findings revealed that the AUC values for the LODDS, N staging, and multivariate models improved over time for 1-, 3-, and 5-year OS. Although the AUC values of the N staging model consistently exceeded those of the LODDS model, indicating higher predictive accuracy, both models exhibited AUC values below 0.7, reflecting moderate accuracy. In contrast, the multivariate model demonstrated superior AUC values for predicting 1-, 3-, and 5-year OS compared to both the LODDS and N staging models, with AUC values for the 3- and 5-year OS reaching or exceeding 0.7. Therefore, we conclude that the multivariate model exhibited the highest accuracy among the three models. Furthermore, a multivariate model for CSS was developed, demonstrating AUC values greater than 0.7 for 1-, 3-, and 5-year CSS, which indicates high predictive accuracy (Fig. 3).

### Visualization of prognostic models

**Fig. 4:**
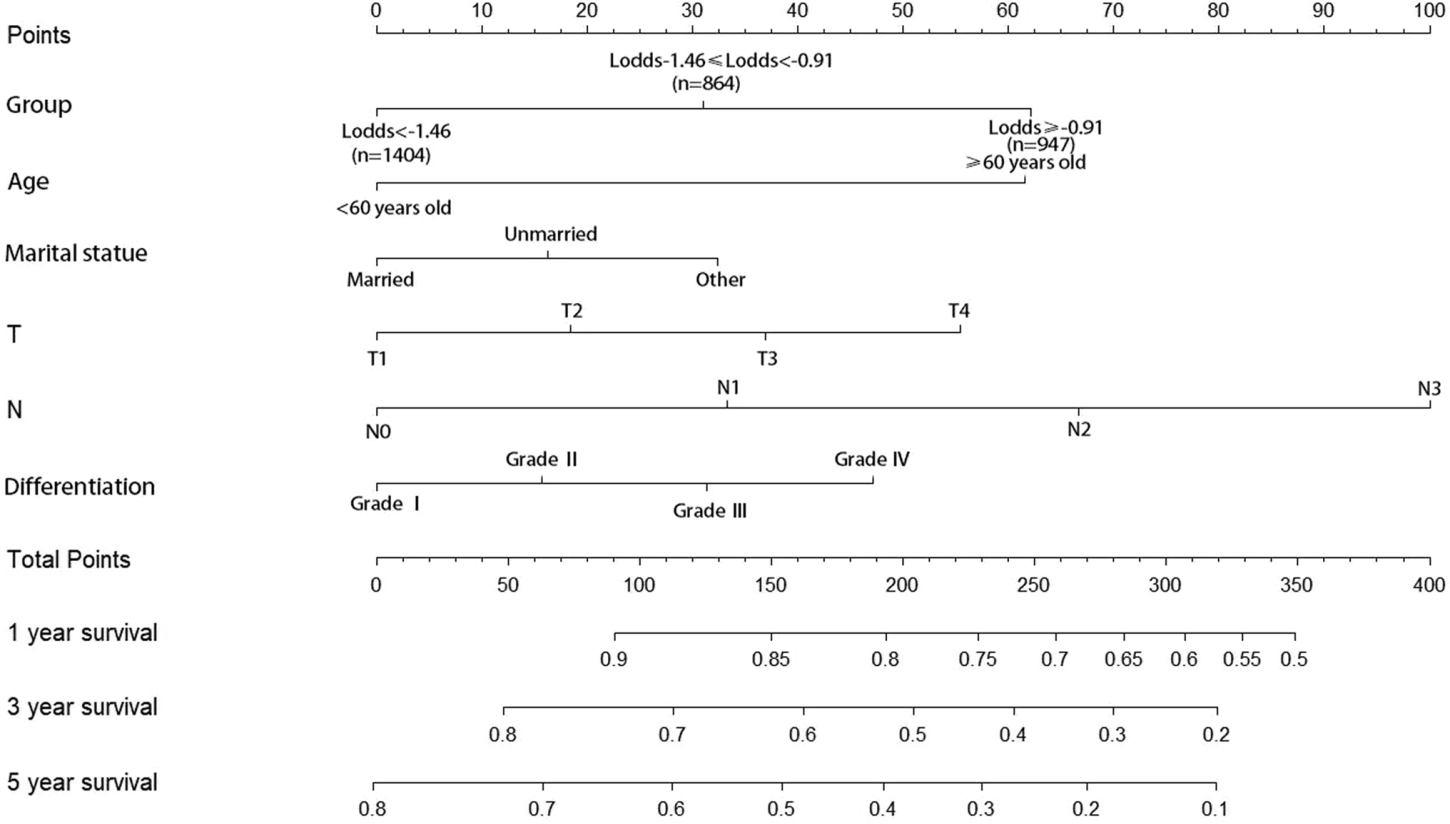
nomogram of 1-, 3-, and 5-year OS for multivariate models (based on LODDS, age, marital status, T stage, N stage, and histological grade

We visualized the multivariable model using a nomogram. As illustrated in Fig. 4, the nomogram assigns predicted values to each variable based on corresponding points, which are subsequently summed to yield a total score. This total score correlates with the scale at the bottom, allowing us to derive the probabilities of OS at 1 year, 3 years, and 5 years. The nomogram for OS shows that the N stage is the most influential factor in predictions, followed by LODDS staging and age.

## Discussion

Accurate tumor staging is crucial for guiding treatment selection, management, and prognosis in patients. LODDS has demonstrated superior prognostic prediction in a wide range of tumors, but its predictive efficacy for postoperative prognosis in laryngeal carcinoma has not been fully evaluated. To our knowledge, this is the first long-term survival prediction model for laryngeal cancer incorporating both LODDS and N staging. Survival and regression analyses in this study showed LODDS as a significant predictor of postoperative prognosis in patients with laryngeal cancer, and a multivariate model combining LODDS and N-staging had the best predictive performance by model comparison.

The AJCC classification system evaluates tumor lymph node metastasis solely based on the anatomical location and size of metastatic lymph nodes. Consequently, the quantitative information provided is often insufficient and does not accurately reflect the specific metastatic conditions or the number of affected lymph nodes. To address this limitation, novel prognostic indicators have been continuously developed. In addition to the LODDS selected for this study, other metrics, such as the NPLN, the metastatic lymph node ratio (MLNR), and the positive lymph node ratio (pLNR), are confirmed as independent predictors of the prognosis for pancreatic, medullary thyroid, and esophageal cancers. This has led to the emergence of numerous prognostic prediction models based on these metrics [16–18]. This study found that both LODDS and N-stage were independent prognostic predictors of survival in patients with laryngeal cancer, which is the same as Wang and Zhang’s study. Compared with Wang's study, we chose the SEER database, which has a larger sample size, thus improving the reliability of the study [19]. Zhang cited several variables and finally chose the NPLN joint LODDS model as the best prognostic model. This model had a C-index of 0.7 or less, which is still less accurate. In addition, their study excluded patients with N0 in N-stage, making the application of this model somewhat limited [20].

Research suggests that LODDS, MLNR, NPLN, and pLNR offer greater prognostic accuracy than traditional N staging for solid tumors, including lung and pancreatic cancer. However, their use in laryngeal cancer research remains underexplored. In this investigation, we developed prognostic prediction models based on LODDS and N staging. The AUC values for the LODDS model at 1, 3, and 5 years were lower than those for the N staging model. Consequently, this study posits that the evaluative value of N staging in laryngeal cancer is superior to that of LODDS, which is further corroborated by the longer length of the N staging in the constructed nomogram compared to LODDS groupings. The SEER database lacks specific data regarding the extent of neck lymphadenectomy, and there are currently no established guidelines in the academic community addressing this issue for laryngeal cancer. The extent of lymphadenectomy performed during surgery is primarily determined by the clinical experience of the operating surgeon. Therefore, we hypothesize that the obtained NPLN values may be underestimated due to selective neck lymphadenectomy, which could adversely affect the resultant LODDS values. Additionally, patients receiving neoadjuvant therapy may undergo a reduced extent of neck lymphadenectomy, further affecting the accuracy of LODDS calculations. This discrepancy may explain the differing performance of LODDS in laryngeal cancer compared to other tumors. The AUC values for both the LODDS and N staging models were relatively low, indicating that they may lack the necessary accuracy for effectively predicting the prognosis of patients with laryngeal cancer.

This study focused on patients who underwent surgical intervention. Although glottic laryngeal carcinoma has the highest incidence, accounting for approximately 60% of cases, its proportion among surgical patients in this study was lower than that of supraglottic carcinoma. This discrepancy may arise because early-stage glottic laryngeal carcinoma usually manifests as hoarseness. due to anatomical differences among various types of laryngeal cancer, the likelihood of early lymph node metastasis is lower. Consequently, the early detection rate of glottic laryngeal carcinoma is higher [21]. Some studies suggest that for early-stage laryngeal malignancies, both surgical treatment and radiotherapy yield comparable oncological and functional outcomes [22–24]. Consequently, patients with early-stage laryngeal carcinoma may opt for radiotherapy alone. This choice may contribute to the observed difference in the ratio of glottic laryngeal carcinoma cases included in this study compared to real-world data. Patients with elevated LODDS stages were more likely to undergo surgical treatment and receive adjuvant radiotherapy. Multivariate Cox regression analysis identified chemotherapy and radiotherapy as independent risk factors for OS and CSS, respectively.. Postoperative adjuvant radiotherapy has been shown to improve survival and local control rates (LCR) in patients with laryngeal cancer. [25]. However, patients undergoing radiotherapy may experience treatment-related complications, including skin damage, xerostomia, oropharyngeal pain, and radiation necrosis of the larynx [26], which increase the risk of preoperative complications such as pharyngeal leaks. While radiotherapy may improve CSS, these complications can negatively impact the overall prognosis. Neoadjuvant chemotherapy has been suggested to preserve laryngeal function, reduce the need for radical neck dissection, mitigate dysfunction associated with such surgery, and ultimately improve postoperative survival and quality of life in patients with laryngeal cancer. [27–29]. However, conflicting evidence exists regarding its benefits. [30]. The findings of this study may serve as supporting evidence that adjuvant therapy has a favorable prognosis for laryngeal cancer patients undergoing surgery.

It is important to acknowledge that this study has several limitations. Unlike N staging, which can be evaluated preoperatively in patients not undergoing surgical treatment through modalities such as PET-CT, enhanced CT, and lymph node ultrasound, LODDS require complete surgical resection and postoperative pathological specimens for accurate assessment. Consequently, this prognostic model is not applicable for preoperative evaluations or for patients receiving only non-surgical treatments. Although there is no specific guideline specification for the extent of cervical lymph node dissection in surgical treatment, it is clear that patients with cT1N0 are generally not suitable for cervical lymph node dissection. This may introduce bias in the application of the LODDS model for this patient group. Furthermore, this study exclusively sourced its data from the SEER database. As a retrospective analysis, our data may be subject to potential selection bias. The SEER database does not provide detailed patient histories or information on critical risk factors, including smoking, excessive alcohol consumption, opioid use, and Epstein-Barr virus (EBV) infection. Furthermore, it lacks data on lymph node invasions, such as lymphovascular embolization, perineural infiltration, extracapsular extension. the absence of specific protocols for non-surgical treatments represents a significant limitation of this research. Additionally, the SEER database contains data exclusively from patients in the United States, which may limit the model’s predictive performance in other regions due to variations in treatment preferences, expertise levels in radiotherapy and chemotherapy, and differences in ethnic demographics. Future studies should prioritize developing databases and establishing validation cohorts across diverse regions to enhance predictive accuracy.

## Limitations section

The data presented in this study are exclusively derived from the SEER database, which entails several inherent limitations in its analysis. The dataset may encompass missing or incomplete entries, especially regarding the details of adjuvant treatments, including specific forms and protocols, that may not have been fully recorded. This shortfall could potentially impinge upon the veracity of survival outcome assessments. Research utilizing SEER data is susceptible to selection bias, and the dataset's dimensions and representativeness may circumscribe the extrapolation of study results to a wider demographic. Additionally, the lack of corroborative validation studies may undermine the solidity of conclusions extrapolated from SEER data, thus underscoring the need for subsequent research to substantiate these findings.

## Conclusion

We generated a prognostic prediction model integrating N staging and LODDS, demonstrating that N-staging exhibits greater predictive significance compared to LODDS within this framework. This model demonstrated favorable predictive performance for 1-year, 3-year, and 5-year OS and CSS in laryngeal cancer patients who underwent surgical intervention. It aims to assist clinicians in assessing patient prognosis and providing individualized treatment recommendations.

## Data Availability

The data used in this study are available from the Surveillance, Epidemiology, and End Results Program (SEER) database (Incidence SEER 17 Registries Nov 2021 Sub) at https://seer.cancer.gov/data-software/documentation/seerstat/nov2021/
